# Adherence of *Staphylococcus aureus* to Dyneema Purity^®^ Patches and to Clinically Used Cardiovascular Prostheses

**DOI:** 10.1371/journal.pone.0162216

**Published:** 2016-09-01

**Authors:** Amir Basir, Paul Gründeman, Frans Moll, Joost van Herwaarden, Gerard Pasterkamp, Reindert Nijland

**Affiliations:** 1 Department of Experimental Cardiology, UMC Utrecht, Utrecht, the Netherlands; 2 Department of Vascular Surgery, UMC Utrecht, Utrecht, the Netherlands; 3 Department of Medical Microbiology, UMC Utrecht, Utrecht, The Netherlands; 4 Laboratory of Phytopathology, Wageningen University, Wageningen, The Netherlands; Laurentian, CANADA

## Abstract

Various materials that are used for vascular and heart valve prostheses carry drawbacks: some require anticoagulant drugs or have moderate durability; others are not suitable for endovascular treatment. These prostheses are associated with bacterial infections. A material potentially suitable for prostheses is Dyneema Purity^®^, made of ultra—high-molecular-weight polyethylene. Dyneema Purity^®^ fibers are very thin, flexible, resistant to fatigue and abrasion, and have high strength. *S*. *aureus* adherence to Dyneema Purity^®^ was tested and compared with currently used cardiovascular prostheses. We compared adhesion of *S*. *aureus* to Dyneema Purity^®^ (1 *membrane-based* and 1 *yarn-composed* patch) with 5 clinically used *yarn-composed* polyester and *membrane-based* expanded polytetrafluoroethylene patches. Patches were contaminated with *S*. *aureus* bacteria and bacterial adherence was quantified. *S*. *aureus* adherence was also visualized in flow conditions. Overall, bacterial adherence was higher on *yarn-composed* prosthesis materials, with a rough surface, than on the *membrane-based* materials, with a smooth surface. Adherence to Dyneema Purity^®^ materials was non-inferior to the currently used materials. Therefore, patches of Dyneema Purity^®^ might be attractive for use in cardiovascular applications such as catheter-based heart valves and endovascular prostheses by their good mechanical properties combined with their noninferiority regarding bacterial adhesion.

## Introduction

Infections of heart valve prostheses or vascular grafts are serious complications. Most of these infections are caused by *Staphylococcus aureus* and *S epidermidis* bacteria. Prosthetic valve endocarditis (PVE) is a grave medical entity with potentially fatal results. This disease encompasses a rather small but, at the same time, vital part of infective endocarditis incidents that is likely to be augmented in the future. Early PVE (i.e., during the first year after implantation) occurs in 1% to 4% of valve recipients, whereas late PVE (i.e., after 12 months of implantation) occurs in approximately 1% per year afterward. The type of prosthetic valve (mechanical vs bioprosthetic) seems not to have an effect on the rate of the development of infective endocarditis and is comparable in both groups at 0.8 cases per year of follow-up [1]. In early PVE, *S aureus* predominates (36%), followed by coagulase-negative staphylococci (17%) and fungi (9%), and almost 17% are culture-negative [1]. At 2 months postoperatively, *S aureus* and coagulase-negative staphylococci account for 18% to 20% of cases each, and viridans streptococci and enterococci are implicated in 10% to 13% of incidents each [2].

The offending agents in late PVE episodes are the same as those causing native valve endocarditis (NVE). A recent multinational prospective cohort study found staphylococci were the causative pathogen in 42% and streptococci in 40% of NVE cases. A cardiac complication will occur in 20% to 40% of PVE patients during the course of the disease. The high frequency of invasive infection results in higher rates of new or changing murmurs, heart failure, and new electrocardiographic conduction disturbances in patients with PVE than in those with NVE. [1] Neurologic complications, such as stroke, meningitis or brain abscess, cerebral hemorrhage, encephalopathy, and seizures occur in 20% to 40% of PVE cases, and the incidence of clinically overt arterial emboli is 40% [3].

Prosthetic vascular grafts (PVGs) are used widely for treatment of dilated or obstructive vascular diseases. Infection rates of PVGs vary between 0.7% and 7%, resulting in amputation of a limb in 50% and death in 25% to even up to 75% of the cases [4,5]. The 5-year survival rate of patients with PVG infection is less than 50% [5]. As described by Hasse *et al*., *S aureus* and the coagulase-negative *S epidermidis* together account for almost 80% of the PVG infections [6]. Within the *Staphylococci*, the beta-lactam—resistant strain of *S aureus*, methicillin-resistant *S aureus* (MRSA), is the most frequent bacterium causing infection of the prosthesis [6,7].

Two prospective studies performed by Naylor *et al*. showed an infection rate of 53% due to *S aureus*, of which MRSA was the pathogen in 62%. Because MRSA is difficult to treat, MRSA-positive patients have a significantly higher risk of death or amputation than MRSA-negative patients [8]. Currently, polyester (polyethylene terephthalate [PET]), also known as Dacron^®^ fabrics, are used extensively in cardiovascular applications, including vascular prostheses, heart valve sewing cuffs, and annuloplasty rings [9]. Dacron^®^ and Gore-Tex^®^ (expanded polytetrafluoroethylene [ePTFE]) vascular grafts have been successful in bypassing and patching of obstructed blood vessels of large and medium diameters [10].

Here we focus on patches of Dyneema Purity^®^ fibers and membrane. Dyneema Purity^®^ materials are made from ultra—high-molecular-weight polyethylene. Dyneema Purity^®^ fibers are approximately twice as strong as steel, are flexible, and are resistant to fatigue and abrasion [11]. Dyneema Purity^®^ membrane is a highly porous thin film. Prostheses made from this material hold promise for use in the minimally invasive treatment of vascular diseases by reducing the profile of the stent graft, including the delivery systems. Heart valves made of Dyneema Purity^®^ fibers could possibly provide a solution for many patients because of the expected prolonged durability and suitability for endovascular implantation. Dyneema Purity^®^ fibers have already been used in cardiovascular applications, such as reinforcement of balloon catheters, and in orthopaedics for anterior cruciate ligament repair [12]. Sutures of Dyneema Purity^®^ fibers are the gold standard for rotator cuff repair [13,14]. Heart valves and vascular constructs of Dyneema Purity^®^ materials would be the first applications where the permanent use with constant contact with blood is considered.

In our previous *in vitro* hemocompatibility studies, patches of Dyneema Purity^®^ fibers were non-inferior in platelet aggregation and coagulation activation compared with currently used cardiovascular prostheses [15]. We tested patches of Dyneema Purity^®^ fibers and membranes to evaluate whether a poor performance regarding bacterial adhesion and infection annuls the beneficial properties of these fibers.

Because *S aureus* is a common pathogen for vascular and valvular infections, the adherence of this bacterium on patches was tested. Bacterial biofilm formation is involved in most, if not all, PVE cases [16]. *S aureus* is well capable of growing in biofilms [6,17], which are structured microbial communities that grow on surfaces and surround themselves with a hydrated polymeric matrix [18,19]. As such, biofilms create their own protective shield, giving bacteria the opportunity to survive high temperatures and antibiotics [20,21]. As a result of this protection, the virulence of the pathogens increases. The shield prevents interference from the immune system, with symptomatic inflammation as a result [20–22]. The formation of a biofilm starts with the initial adherence of a bacterium to a surface such as a prosthesis; thus, surface properties that limit this initial adherence are important to prevent biofilm formation.

This study assessed the adherence of *S aureus* on *yarn-composed* and *membrane-based* patches of Dyneema Purity^®^ materials compared with clinically used polyester and ePTFE prostheses in 3 different experiments.

## Materials and Methods

### Serum preparation

Blood was drawn from healthy volunteers, and serum was obtained after pooling the sera of at least 20 healthy donors and stored until use at –70°C. Informed written consent was obtained from all donors and was provided in accordance with the Declaration of Helsinki. Approval was obtained from the Medical Ethics Committee of the University Medical Center Utrecht (Utrecht, the Netherlands).

Patches of Dyneema Purity^®^ materials were manually cleaned before testing by reversed osmosis water with 10 mL/L alkaline detergent neodisher^®^ MediClean forte (Dr. Weigert Nederland BV, Assen, The Netherlands), rinsed with sterile water, disinfected with 70% ethanol, and sterilized with 100% ethylene oxide at 370 mbar pressure and 50°C for 4 hours with an aeration time of 48 hours. The control patches were not cleaned and sterilized with this method. They are used as commercially available in sterile packaging.

### Patches of *Dyneema Purity*^®^ materials and control patches

*S aureus* adherence on 2 different patches of Dyneema Purity^®^ materials were measured: (1) Dyneema Purity^®^ fibers patch (yarn-based), a woven patch of Dyneema Purity^®^ dtex10 fibers, and (2) Dyneema Purity^®^ membrane patch, which consists of the Dyneema Purity^®^ membrane (membrane-composed). These materials were compared with 4 clinically used cardiovascular prostheses: (1) Bard^®^ DeBakey^®^ Knitted Polyester Fabric, a permeable fabric used for patch graft angioplasty and repair of intracardiac defects; (2) Bard^®^ DeBakey^®^ Woven Polyester Fabric, a thin, low-permeability polyester fabric used in outflow tract repairs, patch graft angioplasty, and septal defects; (3) IMPRA^®^ ePTFE Cardiovascular Patch applications, which include peripheral vascular reconstructions and cardiac and vascular repairs; and (4) Gore-Tex^®^ Stretch Vascular Graft Standard Wall, which is tubular shaped and used for different purposes in angiographic access.

Of these 6 patches, the Dyneema Purity^®^ fibers patch, Bard^®^ DeBakey^®^ Knitted, and Woven Polyester Fabric do not have a smooth surface because as they are fabric (e.g. *yarn-composed*). The other three patches—Dyneema Purity^®^ membrane patch, IMPRA^®^ ePTFE Cardiovascular Patch, and Gore-Tex^®^ Stretch Vascular Graft Standard Wall—are *membrane-based* and have a smooth surface.

### Incubation of patches with *S*. *aureus*

All patches were cut to the same size of 1.0x1.0cm, placed in a well of a 24-well plate, and covered by 1 mL of 20% human pooled serum in phosphate-buffered saline (PBS) containing a derivative of the high-virulence community-acquired strain of MRSA, *S aure*us MW2 + pCM29 (green fluorescent protein [GFP]+, chloramphenicol resistant) at optical density (OD)_595_ 0.05 (~1.10^7^ bacteria/mL) (from an overnight culture of *S aureus*, grown until early log-phase [OD_595_ ~0.5] and diluted to OD_595_ 0.05) [23]. The human pooled serum was a mixed human serum obtained from 20 to 25 volunteer donors.

The patches were incubated at 37°C for 10, 30, and 90 minutes with gentle agitation by orbital shaking at 300 rpm. After incubation, the patches were transferred under sterile conditions to a 15-mL tube containing 3 mL of sterile PBS and were rinsed by gentle agitation to remove the nonadherent bacteria. The rinse was repeated 3 times. To quantify the bacterial adhesion on the patches, the adhered bacteria were removed by transferring the patches to a new tube containing 3 mL of PBS and DNaseI (0.1 mg/mL), vortexing vigorously, incubating for 15 minutes at 37°C, vortexing vigorously again, sonicating for 60 seconds in a sonication bath (Branson 2510 E-MT, Branson Ultrasonics, Danbury, CT, USA), and by storing on ice until all samples were obtained. DNaseI was added to the PBS to break down the extracellular DNA matrix that allowed *S aureus* to adhere to the materials. Materials were stored on ice to prevent additional growth and cell division of *S aureus*. We verified that the mild enzymatic and physical treatment used to remove the adherent bacteria from the materials tested did not affect their viability (data not shown).

The remaining bacteria on the patches were removed and quantified by 2 different methods: (1) by plating a serial dilution and counting the colony forming units (CFUs) on plate and (2) by monitoring the outgrowth time as a value for initial inoculum by measuring the OD in time. Also, the *S aureus* adherence during flow conditions was visualized.

### Bacterial enumeration by CFU counting

**Fig 1:**
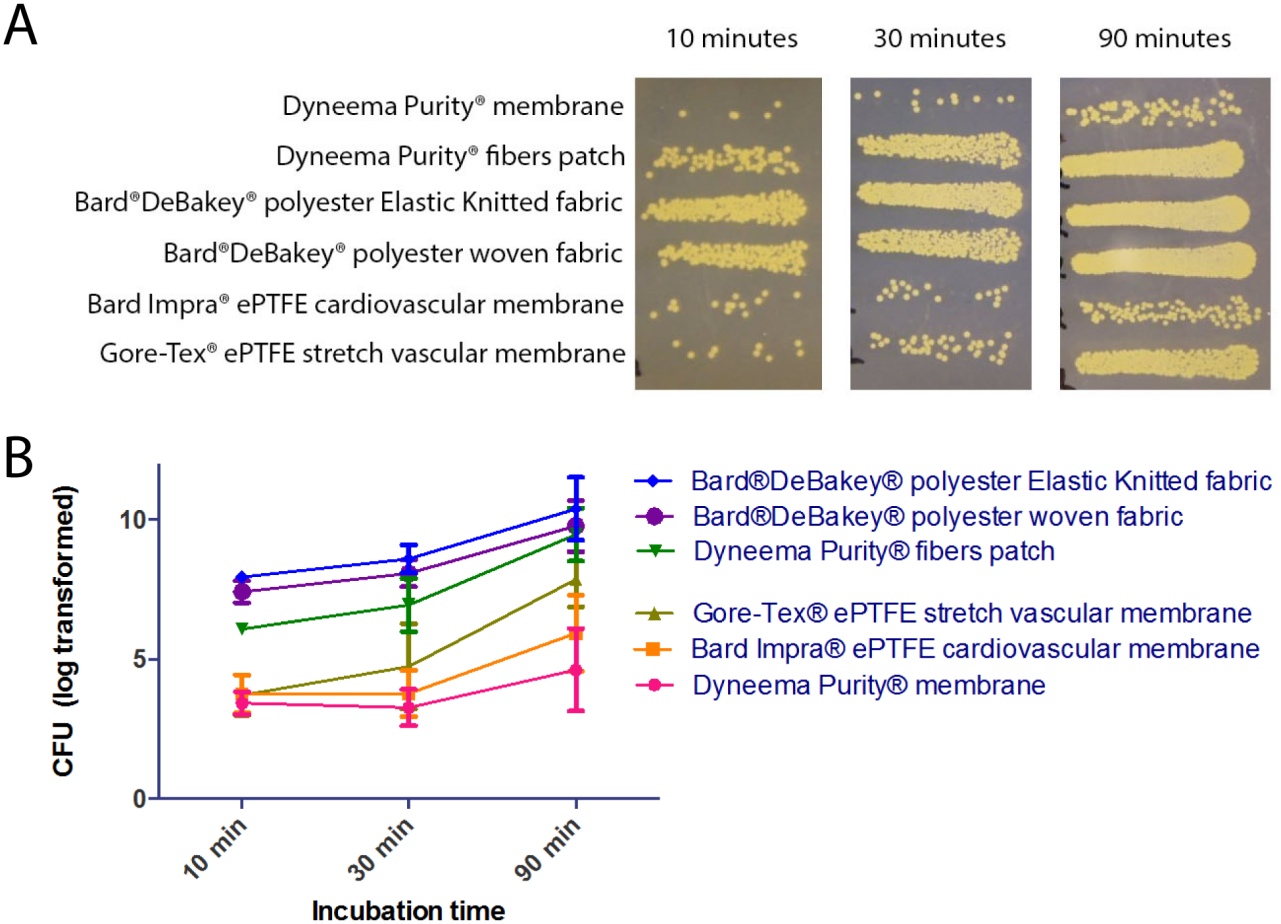
Bacterial adhesion to the patches of Dyneema Purity^®^ materials and the control patches as measured by colony forming unit (CFU) counts. A: Representative example of CFUs on agar plates after 10, 30, and 90 minutes of incubation of the 6 different *yarn-composed* and *membrane-based* patches: Dyneema Purity^®^ membrane; Bard Impra^®^ ePTFE Cardiovascular Patch; Gore-Tex^®^ Stretch Vascular Graft Standard Wall; Dyneema Purity^®^ fibers patch; Bard^®^DeBakey^®^ Elastic Knitted and woven Polyester Fabric. B: Log-transformed average CFU of 3 experiments after 10, 30, and 90 minutes incubation. The error bars represent the standard deviation.

For bacterial enumeration on agar plates, the adherent bacteria collected from the patch treatment described above were serially diluted using a 96 well microtiter plate. Using a multichannel pipette, 5 μL of each patch supernatant sample with an identical dilution was pipetted on one side of the agar plate. The plate was tilted from side to side in order to run the droplets across the plate, allowing enumeration of 5 samples in identical conditions on 1 plate. This was performed directly after the 90-minutes sample was obtained, and a fresh nutrient agar plate was used for each dilution. Plates were incubated overnight for colony development and CFUs were counted from the appropriate dilution (Fig 1).

### Bacterial enumeration by measuring the outgrowth time

**Fig 2:**
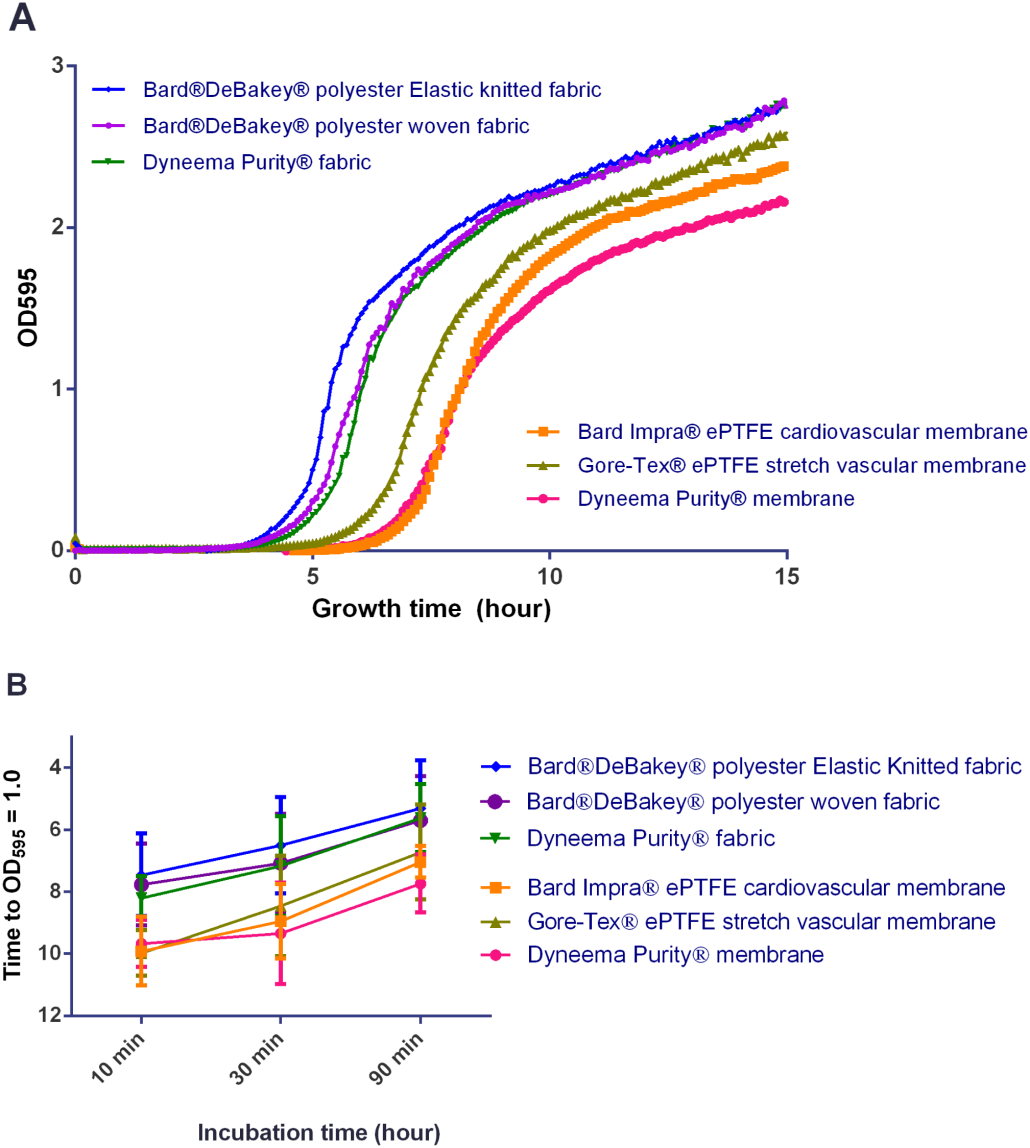
Bacterial adhesion to the tested patch materials as quantified by outgrowth time. A: Representative example of growth curves as determined in plate reader, after inoculation with *S*. *aureus* adhering to patch materials after 30 minutes incubation. B: Growth time to OD_595_ = 1.0 (average ± standard deviation) after inoculation with *S*. *aureus* adhering to patch materials after inoculation with *S*. *aureus* after 10, 30, and 90 minutes of incubation.

To determine the adhered bacteria on the patches, the time required for the initial inoculum density to reach OD_595_ of 1.0 (mid-log for this strain/plate reader combination) was measured using a Fluorstar Omega microplate reader (BMG Labtech, Germany) (Fig 2). The inoculum was the same as used for the CFU plating, and therefore, contained bacteria that were adhered and subsequently released from the patches. Next, 10 μL of the undiluted supernatant was added to 150 μL of Lysogeny broth (LB) medium in a 96-well plate, and growth of the bacteria was measured every 5 minutes up to 15 hours. There is an inverse relation between the number of viable bacteria in the initial inoculum and the time required to reach OD_595_ of 1.0. Using this number provided an independent measurement to compare the different patches. All initial inocula were below the detection limit of the plate reader OD sensor. After a certain amount of duplications, culture density reached the detection limit and a growth curve could be observed.

### Adherence and microcolony formation in flow conditions

To study the effect of shear stress on bacterial adherence to the different patches, perfusion experiments were performed in a flow cell, as described by Arenas *et al*., that was mounted on the stage of a Leica SP5 microscope [24]. For this experiment, the adherence on *yarn-composed* Dyneema Purity^®^ fibers patch was compared with *yarn-composed* Bard^®^DeBakey^®^ Elastic Knitted Polyester Fabric and the *membrane-based* Bard Impra^®^ ePTFE Cardiovascular Patch. Patches were cut in strips of the same size (3 × 10 mm) and were glued at either side to the bottom of a 3-channel flow cell (DTU Copenhagen) using silicon glue (Scrintec 802, Carl Roth GmbH, Karlsruhe, Germany). Culture conditions were kept at a temperature of 37°C, and a flow rate of 250 μL/min per channel was used for LB growth medium supplemented with 5 μg/mL chloramphenicol. GFP-labeled *S aureus* MW2 was inoculated in the bubble trap of the flow cell with a starting OD_600_ of 0.02. A relative constant inflow of the number of *S aureus* cells was provided due to equilibrium between the bacterial replication in the bubble trap and the dilution rate by the inflowing medium.

Adherence and subsequent microcolony and biofilm formation was visualized using time-lapse microscopy, and photographs were made every 7 minutes for 5 hours using HCX PL APO CS 10.0 × 0.40 DRY objective in both the bright field and the GFP channel. These photographs were taken from the middle of each patch to exclude the effects of *S aureus* adherence due to the potential ravels of the patch strips and to the effects of the silicon glue used for mounting the patch strips in the flow cell.

### Analysis and statistics

For the CFU experiment, unpaired, 2-tailed *t* test, calculated with GraphPad Prism 6.0 software, was used to analyze the differences between the different patch materials. A *P* value of ≤0.05 was considered statistically significant.

MARS software package (BMG Labtech, Germany), and Excel (Microsoft Corp, USA) was used to analyze the outgrowth time in the plate reader experiment.

## Results

Attachment of the bacteria is an essential first step towards biofilm formation and infection. Therefore attachment of MRSA to Dyneema Purity^®^ is compared with five control materials after 90 minutes. The Dyneema Purity^®^ fibers patch and Dyneema Purity^®^ membrane showed equal or less bacterial adherence compared with *yarn-composed* and *membrane-based* controls (Fig 1). After 10 and 30 minutes of incubation, fewer CFUs were observed on the *yarn-composed* patch of Dyneema Purity^®^ fibers (*P* <0.0001) than on the Bard^®^DeBakey^®^ Elastic Knitted Polyester Fabric (*P* = 0.02). After 90 minutes, the differences between the patches for CFU formation were not significant.

For *membrane-based* patches, there were no differences after 10 and 30 minutes. After 90 minutes, fewer CFUs were observed on Dyneema Purity^®^ membrane patch than on the Gore-Tex^®^ Stretch vascular graft (*P* = 0.01).

In general, *yarn-composed* patches had 10 times (Dyneema Purity^®^ fibers patch) to 100 times (Bard^®^ DeBakey^®^ Knitted, and Woven Polyester Fabric) higher adherence than the *membrane-based* patches after 10 minutes (Fig 1). Surprisingly, CFUs from Gore-Tex^®^ Stretch Vascular Graft Standard Wall increased 10-fold after 30 minutes, whereas the CFUs of other patches showed a very small increase (*membrane-composed*) or a 2-fold to 5-fold increase (*yarn-composed*). The observed effect after 90 minutes’ incubation revealed that adherence on most patches was increased 10-fold compared with adherence after 30 minutes.

**Fig 3:**
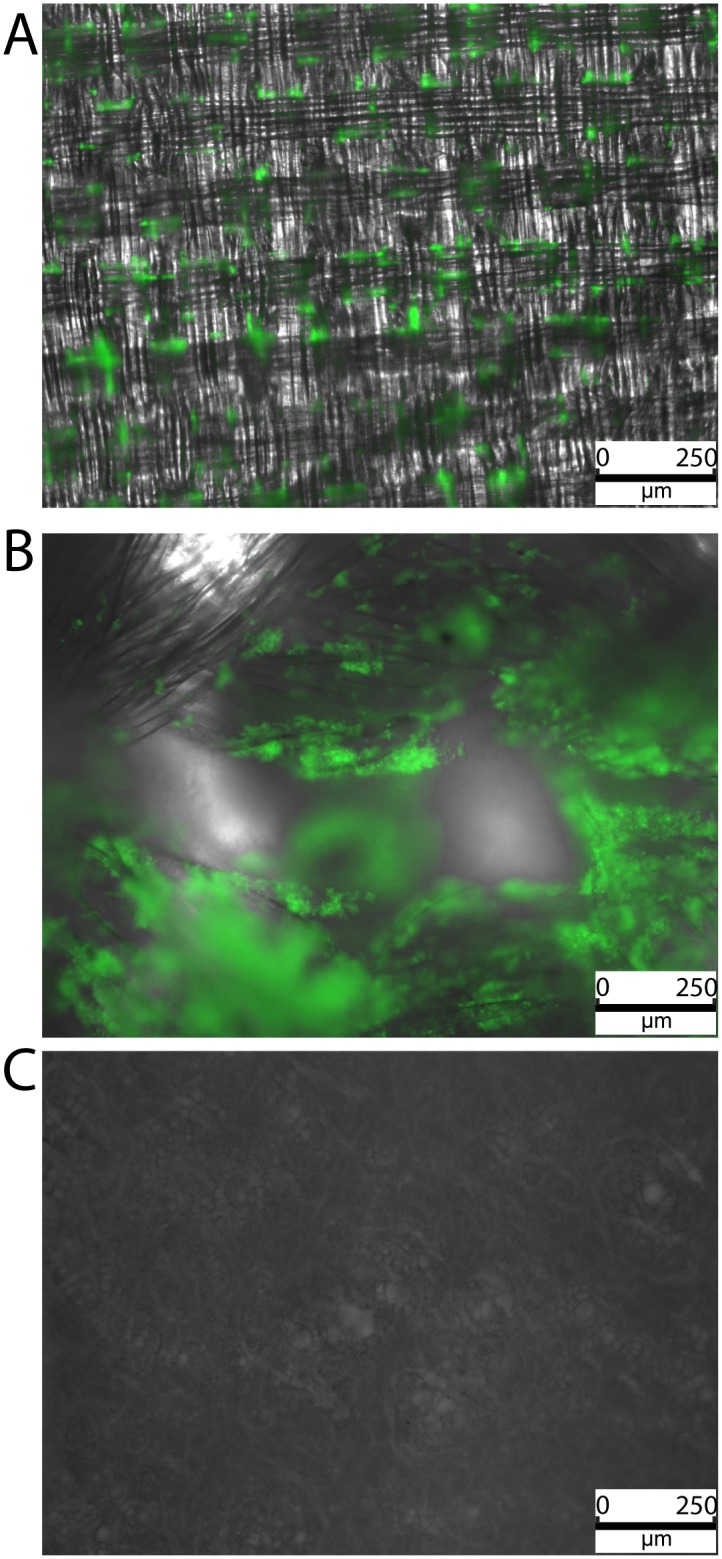
Adherence of GFP-labeled *S aureus* to patch materials under flow conditions, 3.5 hours after start of flow. A: Dyneema Purity^®^ fibers patch. B: Bard^®^DeBakey^®^ Elastic Knitted Polyester Fabric. C: Bard Impra^®^ ePTFE Cardiovascular Patch.

The bacterial enumeration by measuring outgrowth time gave highly similar results to the CFU counting (Fig 3).

### Adherence and microcolony formation in flow conditions experiment

From the time-lapse movies, it is clear that bacteria hardly adhere to the *membrane-based* ePTFE patches (Fig 3C, S3 Movie), whereas the *yarn-composed*, and especially the Bard^®^ DeBakey^®^ Knitted Polyester Fabric (Fig 3B, S2 Movie) with its very rough structure, are much more amenable for adherence of bacteria. We show the time lapse images of tree of the six tested materials, representative for the differences observed in these flow conditions. A complicating factor in analyzing woven vs. knitted *yarn-composed* patches was the thickness of both patches. The flat yarn-composed Dyneema Purity^®^ fibers patch has its surface in a relative small plane, and all adhered bacteria were visible in the time-lapse image. The *yarn-composed* Bard^®^ DeBakey^®^ Knitted Polyester Fabric is much thicker and more porous, which rendered many of the adhering bacteria out of focus. The increase in out-of-focus bacteria could be observed by the increase in the distinct green glow of the overall image (Fig 3; S1, S2 and S3 Movies).

## Discussion

This study assessed bacterial adherence on *yarn-based* (i.e. Dyneema Purity^®^ fibers patch) and on *membrane-composed* (i.e. Dyneema Purity^®^ membrane patch) materials compared with 4 commonly used *yarn-composed* and *membrane-based* control materials in cardiovascular surgery. These were the Bard^®^ DeBakey^®^ Knitted Polyester Fabric, the Bard^®^ DeBakey^®^ Woven Polyester Fabric, Bard Impra^®^ ePTFE Cardiovascular Patch, the Gore-Tex^®^ Stretch Vascular Grafts Standard Wall.

Patches were incubated with a derivative of the high-virulence community-acquired strain of MRSA, *S aure*us MW2. This allowed for testing of initial adherence to the membranes, which is the first and most important step in establishing an infection. These *in vitro* conditions are not suitable for testing for extended periods because the conditions in the experiment are very beneficial for bacterial growth and biofilm formation on practically any material. In our study, thick biofilms had formed on all materials tested after 24 hours, (data not shown).

The main findings of our study were that Dyneema Purity^®^ yarn-based patches are non-inferior to yarn-based control patches and that Dyneema Purity^®^ membrane patches are non-inferior to membrane-based control patches regarding bacterial adherence. Lower adherence was demonstrated for *yarn-composed* Dyneema Purity^®^ fibers patches after 10 and 30 minutes compared with the *yarn-composed* Bard^®^DeBakey^®^ Elastic Knitted Polyester Fabric control patch. On *membrane-based* patches, lower adherence was observed on Gore-Tex^®^ Stretch vascular graft patch after 10 minutes. At 30 minutes there were fewer CFUs for Dyneema Purity^®^ membrane patches compared with membrane-based test patches. It is remarkable that, at 90 minutes of incubation fewer colonies were obtained for Dyneema Purity^®^ membrane patches in comparison with Gore-Tex^®^ Stretch vascular graft. However, when compared to the Bard Impra^®^ ePTFE Cardiovascular patches adherence is very similar. The differences between these two materials are likely to be caused by the mechanical properties or the coating of these materials.

The results obtained with the outgrowth experiments were highly similar to the data from the CFU counting, indicating that both methods are suitable to determine inoculum concentration and that the CFUs identified most likely did not contain large aggregates after the treatment to release them from the patches after adherence. In cases where aggregates (such as biofilm fragments) are still present, the outgrowth experiments are highly valuable. A single aggregate would result in a single CFU, but nevertheless could consist of large amounts of viable cells, which would be detected in the outgrowth experiment by a shortened outgrowth time. Therefore, the combination of both methods used for determining inoculation concentration provides insight in the presence of multicellular aggregates.

The formation of bacterial biofilms is a major issue during infections such as PVE [16]. Because the formation of a biofilm starts with the adherence of single bacteria to a substrate [19], the aim of this study was to analyze the adherence of *S aureus*, the predominating pathogen in bloodstream infections, on patches of Dyneema Purity^®^ materials and compare these results with currently used cardiovascular prostheses. The methods used in this experiment allowed a standardized way to study the material and the structural properties of different patches in relation to *ex vivo* blood.

To assess bacterial adhesion on *yarn-composed* and *membrane-based* patches of Dyneema Purity^®^ materials, we compared them with 2 *yarn-composed* prostheses that had a relatively rough surface and with 2 *membrane-based* prostheses that had a relatively smooth surface (Table 1). We noticed that, unsurprisingly, more bacteria adhered to the *yarn-composed* prostheses than to the *membrane-based* prostheses. The different structure of the patches combined with the chemical properties of the different materials likely played a major role here [25,26]. The higher bacterial adherence on the *yarn-composed* prostheses with a rough surface than on the *membrane-based* prostheses with a smooth surface is probably because the bacteria got trapped between the wires (“yarns”) of the *yarn-composed* prostheses and because the total available surface for adherence is much larger for *yarn-composed* prostheses.

**Table 1:** Specification of the *yarn-composed* and *membrane based* Dyneema Purity^®^ patches and other *yarn-composed* and *membrane-based* control patch materials.

Material	Specification
Dyneema Purity^®^ membrane patch	*Membrane-based*
Bard Impra^®^ ePTFE Cardiovascular Patch	*Membrane-based*
Gore-Tex^®^ ePTFE Stretch Vascular Graft	*Membrane-based*
Dyneema Purity^®^ fibers patch (woven Dyneema Purity^®^ TG dtex10 fibers)	*Yarn-composed*
Bard^®^DeBakey^®^ Polyester Elastic Knitted Fabric	*Yarn-composed*
Bard^®^DeBakey^®^ Polyester Woven Fabric	*Yarn-composed*

The favorable mechanical properties of Dyneema Purity^®^ fibers combined with the non-inferiority of these patches in bacterial adhesion compared with the currently used cardiovascular prostheses makes *yarn-composed* and *membrane-based* patches of Dyneema Purity^®^ materials attractive for use in endovascular cardiovascular applications such as heart valves and vascular prostheses. These very thin yet very strong fibers enable reducing the size of heart valves and vascular prostheses, making them more suitable for use in endovascular treatment. In our previous *in vitro* hemocompatibility studies, patches of Dyneema Purity^®^ fibers were non-inferior in platelet aggregation and coagulation activation compared with currently used cardiovascular prostheses [15]. Although further research is necessary to test whether these *in vitro* results will hold true *in vivo*, the non-inferior properties regarding bacterial adhesion and hemocompatibility make Dyneema Purity^®^ fibers a highly promising material for vascular and hearth valve prosthesis.

## Supporting Information

S1 Movie Time-lapse of adherence of GFP-labeled *S aureus* to Dyneema Purity^®^ fibers patch under flow conditions. (MOV)

S2 Movie Time-lapse of adherence of GFP-labeled *S aureus* to Bard^®^DeBakey^®^ Elastic Knitted Polyester Fabric under flow conditions. (MOV)

S3 Movie Time-lapse of adherence of GFP-labeled *S aureus* to Bard Impra^®^ ePTFE Cardiovascular Patch under flow conditions. (MOV)
